# Antioxidative, Glucose Management, and Muscle Protein Synthesis Properties of Fish Protein Hydrolysates and Peptides

**DOI:** 10.1021/acs.jafc.4c02920

**Published:** 2024-09-19

**Authors:** Niloofar Shekoohi, Brian P. Carson, Richard J. Fitzgerald

**Affiliations:** † Department of Biological Sciences, University of Limerick, V94 T9PX Limerick, Ireland; ‡ Department of Physical Education and Sport Sciences, Faculty of Education and Health Sciences, University of Limerick, V94 T9PX Limerick, Ireland; § Health Research Institute, University of Limerick, V94 T9PX Limerick, Ireland

**Keywords:** fish protein hydrolysates, bioactive peptides, antioxidant, glycaemic management, muscle health

## Abstract

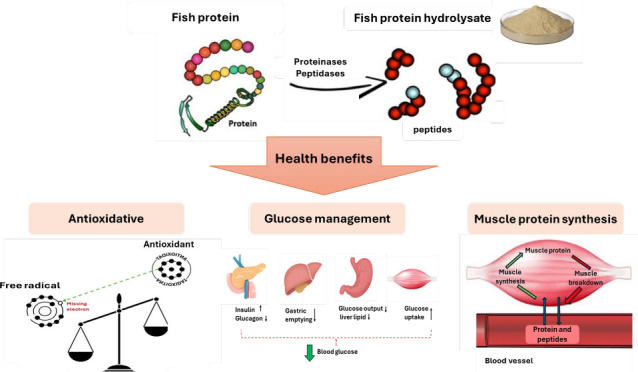

The marine environment is an excellent source for many physiologically active compounds due to its extensive biodiversity. Among these, fish proteins stand out for their unique qualities, making them valuable in a variety of applications due to their diverse compositional and functional properties. Utilizing fish and fish coproducts for the production of protein hydrolysates and bioactive peptides not only enhances their economic value but also reduces their potential environmental harm, if left unutilized. Fish protein hydrolysates (FPHs), known for their excellent nutritional value, favorable amino acid profiles, and beneficial biological activities, have generated significant interest for their potential health benefits. These hydrolysates contain bioactive peptides which are peptide sequences known for their beneficial physiological effects. These biologically active peptides play a role in metabolic regulation/modulation and are increasingly seen as promising ingredients in functional foods, nutraceuticals and pharmaceuticals, with potential to improve human health and prevent disease. This review aims to summarize the current *in vitro*, cell model (*in situ*) and *in vivo* research on the antioxidant, glycaemic management and muscle health enhancement properties of FPHs and their peptides.

## 1 Introduction

The marine environment represents one of the most valuable natural resources on planet earth as it provides food largely in the form of fish and shellfish. It is also an excellent natural resource for many physiologically active compounds due to its extensive biodiversity. This large diversity and the dynamics of the marine ecosystem makes it an ideal reservoir for the identification of new molecules and for the development of marine-derived health enhancing components/ingredients. More than 20,000 marine bioactive compounds have been isolated, however, only a small proportion of these have been thoroughly studied and exploited.^1^ In 2018, the global market for marine-derived compounds was over $10 billion US, which is expected to increase to $22 billion by 2025 at a compound annual growth rate of 11.3% from 2019 to 2025.^2^

Among marine resources, fish proteins possess unique characteristics that make them valuable ingredients in various applications. These proteins can be derived from different fish species, including marine and freshwater, and they exhibit a wide range of compositional and functional properties.^3^ The protein content in raw fish flesh and shellfish varies, typically ranging from 17 to 22% (w/w) and from 7 to 23% (w/w), respectively.^4^ The aquaculture of fish is increasing globally with more than 196 million tons expected to be grown in 2025.^5^ The fish processing industry generates large amounts of coproducts such as heads, skin, trimmings, fins, viscera, frames and sometimes muscle, which are currently either wasted, underutilized, or used to produce low value products such as fishmeal and fish silage. These coproducts may amount to 50% of the whole fish but this can range from 10% to 90%, depending on the fish species and the intended use.^6,7^ There are large volumes of discards on board fishing vessels, which account for 9–15% of global catch, leading to significant levels of underutilized fish resources.^8^ There is an increased focus on these underutilized protein sources due to the worldwide demand for high quality, sustainable protein to support the growing population. Alongside advancements in capturing and processing techniques, the extraction and purification of bioactive compounds such as peptides and amino acids (AAs) from underutilized mesopelagic fish species could offer an economically and nutritionally sustainable strategy for greater utilization of this resource. Fish processing proteinaceous coproducts have, e.g., the potential to be used as sources of essential amino acids (EAAs), collagen/gelatin and enzymes.^9^ The crude protein content of fish processing coproducts ranges between 8 and 35%.^10^ Fish protein coproducts can be used to generate protein hydrolysates and bioactive peptides, increasing their economic value while decreasing their potentially negative environmental impact.^11^ Because fish protein hydrolysates (FPHs) can have an excellent nutritional composition, favorable AA profiles and beneficial biological activities, there has been significant interest in examining their potential industrial applications as functional food ingredients.^12^

Protein hydrolysates are complex mixtures of oligopeptides and free AAs formed by partial or extensive hydrolysis which are generally produced during enzymatic hydrolysis of intact proteins with proteolytic and peptideolytic enzyme activities.^13^ The bioactivity of peptides is related to their AA composition and sequence.^14^ Biologically active peptides may play a crucial role in metabolic regulation and modulation.^15^ They therefore hold potential as functional food ingredients, nutraceuticals and pharmaceutical agents, offering opportunities to enhance human health and prevent diseases. The reported activities of these biopeptides include antihypertensive, opioid agonist or antagonist, immunomodulatory, antithrombotic, antioxidant, anticancer and antimicrobial activities.^15^ Moreover, several peptides have been reported to exhibit multifunctional bioactivities.^16^ FPHs and associated peptides may provide health benefits, consequently, FPHs and their peptides are being promoted as functional food ingredients. This review focuses on the *in vitro*, cell (*in situ*) and *in vivo* evidence concerning the antioxidant, glycaemic management and muscle health enhancement effects of FPHs and their associated peptides.

## 2 Proximate Composition of FPH

The process of producing FPH is crucial for their chemical composition and nutritional value. According to the literature, the *in vitro* hydrolysis of protein substrates using appropriate exogenous proteolytic enzymes is the most widely used process for the production of protein hydrolysates and peptides with desirable biological properties.^17^ Most food proteins contain bioactive peptides that are inactive within the sequence of their parent proteins and can be released by enzymatic hydrolysis, either in the body during gastrointestinal digestion by endogenous proteases or during food processing (e.g., during fermentation) or by proteolytic processes using appropriate exogenous proteases.^13^ The chemical composition of food materials has a significant impact on human health in terms of their ability to supply essential nutrients. Many reports indicate the protein equivalent content of FPHs to range between 60 and 90% (w/w) on a dry weight (dw) basis.^18−25^ The high protein equivalent content reported for FPHs is due to solubilization of proteins during hydrolysis and removal of insoluble solid matter by, e.g., centrifugation.^26,27^ The high protein equivalent content in FPHs demonstrates their potential use as protein supplements for human nutrition. Several studies report the lipid content for various FPHs to be below 5% (w/w dw).^19,21−23,28−30^ A limited number of reports describe lipid contents above 5% (w/w) for FPHs.^24−26^ The low lipid content of FPHs may be due to the removal of lipid with the insoluble protein fraction during centrifugation. Most studies report that hydrolysates from various fish proteins contain moisture levels below 10% (w/w) dw.^19,21−23,26,28,30,31^ The relatively low moisture contents of protein hydrolysates may be related to the type of sample and to the high temperatures employed during the evaporation and spray drying processes employed to stabilize these hydrolysates.^32^ The ash content of FPHs was reported to range between 0.45 and 27% (w/w) dw.^18,21−23,26,30,33,34^ This relatively high ash content of FPHs may, in part, be related to addition of acid or base during the enzymatic hydrolysis process as a means of controlling the pH.

Protein hydrolysates are made up of free AAs and short chain peptides. Table 1 shows the AA composition of some protein hydrolysates prepared from various fish processing protein-rich coproducts. Aspartic acid and glutamic acid were found to be highly abundant in the majority of the reported FPHs. All EAAs and nonessential amino acids (NEAAs) were found in FPHs, although the aromatic amino acids were not found or found at low levels in Alaska pollock frame (APF, derived from frozen backbones) protein hydrolysates.^35^Table 1 also illustrates the high content of branched chain amino acids (BCAAs) in FPHs which may make FPHs a good source for enhancement of muscle health via stimulation of protein synthesis.

**Table 1:** Amino Acid Composition of Fish Protein Hydrolysates (mg/g Protein/Powder) Generated from Different Fish Species Following Hydrolysis Using Alcalase (A) and Flavourzyme 500L (F)^a^

		red salmon (*Oncorhynchus nerka*) hydrolysates (mg/g protein)^167^		herring (*Clupea harengus*) byproduct hydrolysates (mg/g protein)^24^			yellow stripe trevally (*Selaroides leptolepis*) protein hydrolysates (mg/g powder)^168^		brown stripe red snapper (*Lutjanus vitta*) muscle protein hydrolysates (mg/g powder)^20^	
amino acid	blue whiting protein hydrolysates (mg/g powder)^163^	A	F	HBH	HHH	blue whiting protein hydrolysates (mg amino acid/g powder)^86^	A	F	A	F
aspartic acid	85.6	88.3	87.7	93.8	89.2	64.2	95.5	94.0	106.5	107.3
threonine	37	41.9	42.1	39.3	40.4	25.9			51.0	49.1
serine	41.0	46.1	46.2	41.1	45.3	25.5	53.5	54.0	51.5	50.2
glutamic acid	125.0	135.1	134.9	163.4	145.1	94.1	137.7	138.9	162.3	169.2
proline	34.1	65.0	66.8	41.0	58.9	20.2	38.1	38.4	36.6	34.6
glycine	55.2	97.6	102.9	69.8	95.0	27.8	88.7	86.5	74.8	72.1
alanine	54.1	68.4	68.8	70.1	69.4	38.4	94.9	94.6	93.8	95.6
valine	38.9	50.1	45.2	44.7	45.7	27.8	36.1	33.8	51.5	51.5
methionine	19.4	28.8	29.4	33.3	33.1	18.2	25.8	18.7	29.3	29.2
isoleucine	33.5	37.1	36.6	33.2	32.6	27.4	41.4	44.9	41.4	40.7
leucine	57.8	66.9	66.5	78.8	71.2	48.0	83.8	85.8	85.4	86.2
tyrosine	32.3	32.5	30.9	26.3	27.4	18.7	57.0	61.1	23.3	21.4
phenylalanine	28.7	40.7	39.7	34.9	37.2	23.1	26.1	26.6	26.5	24.5
histidine	15.3	23.8	23.0	26.0	18.9	8.6	36.2	29.8	17.1	16.0
hydroxyproline		23.5	25.6			0.0				
lysine	74.4	73.9	72.5	106.6	80.4	53.9	83.5	87.2	95.1	97.6
arginine	59.4	67.7	69.2	70.5	73.0	40.7	35.0	38.8	50.0	50.6
tryptophan	6.5					0.0			2.8	2.9
cysteine	6.1			11.2	11.1	6.5	14.7	15.3	1.2	1.4

a HBH = herring body (only head and gonads were removed) hydrolysates (generated with Alcalase); HHH = herring head hydrolysates (generated with Alcalase)

## 3 Biological Activities of FPH

Peptides obtained from the hydrolysis of food proteins have generated significant attention for their potential applications as nutritional and functional food ingredients. FPHs have been identified as favorable protein sources for human consumption due to their well-balanced AA composition and their beneficial impact on human nutrition.^36^ Recent studies demonstrate that FPHs may have a number of physiological effects beyond their primary role as sources of nitrogen and EAAs.^37^ A detailed search of various academic databases, including PubMed, ScienceDirect, and Google Scholar, using relevant keywords such as “fish protein hydrolysates,” “antioxidant activity,” “fish byproducts” and “bioactive peptides” was performed herein. The search focused on studies to capture recent advancements in the field.

### 3.1 Antioxidant Activity

It is now widely acknowledged that consuming dietary antioxidants is an effective approach to enhance the body’s antioxidant capacity and to counteract the effects of reactive oxygen species (ROS).^15^ The mechanisms by which this occurs include ROS inactivation,^38^ scavenging of free radicals,^39^ chelation of pro-oxidative transition metals^40^ and in the reduction of hydroperoxides.^41,42^ Certain amino acids, such as His, Glu, Asp along with phosphorylated Ser and Thr possess the ability to chelate prooxidative transition metals.^40^ Peptides can possess potent antioxidants activity due to the enhanced stability of the stable molecular structure formed when antioxidants react with a free radical.^15^ The antioxidant potential of a protein or peptide relies on the accessibility of the amino acids therein to prooxidants. Various protein hydrolysates and peptides exhibit potent antioxidant activity.^43^ Many antioxidant hydrolysates and peptides reported in the literature are from fish sources. As an example, longtail cod, cod, and mackerel hydrolysates showed higher antioxidant activity than common antioxidants such as α-tocopherol and butylated hydroxy anisole (BHA).^43^ In another study, the antioxidant activities of protein hydrolysate powders generated from the soluble protein fraction in blue whiting soluble protein hydrolysate (BWSPH) using six commercial enzymes were tested.^44^ Significant variation in the 2,2-diphenyl-1-picrylhydrazyl (DPPH) scavenging capacity was observed across all BWSPHs. Distinct differences in scavenging capability emerged at concentrations of 3.0 mg/mL and higher. The Flavourzyme generated hydrolysate exhibited significantly higher scavenging potential compared to the BWSPHs generated with the other enzyme preparations. Regarding reducing power, the Protamex hydrolysate displayed significantly higher values compared to the other BWSPHs. Moreover, the ferrous chelating ability assay demonstrated a comparable concentration-dependent enhancement in antioxidant capacity for all the BWSPHs.^44^ The antioxidant properties of fish hydrolysates and peptides have primarily been evaluated using *in vitro* assays that measure the scavenging activity of free radicals and ROS. These assays include tests such as the DPPH radical scavenging activity assay, the 2,2′-azino-bis (3-ethylbenzothiazoline-6-sulfonic acid) (ABTS) assay, the oxygen radical absorbance capacity (ORAC), the hydroxyl (OH) radical scavenging activity and the superoxide anion (O2) radical scavenging activity assays. Mammalian cells contain a range of enzyme activities that can effectively hinder or deter the formation of free radicals or reactive species. These include superoxide dismutase (SOD), catalase (CAT) and glutathione peroxidase (GSH-Px). SOD functions by dismutating superoxide radicals, while CAT and GSH-Px break down hydrogen peroxide and hydroperoxides into harmless molecules, namely water (H_2_O) and oxygen (O_2_).^45^ It was reported that a BWSPH exhibited pronounced antioxidant effects both pre- and postsimulated *in vitro* gastrointestinal digestion (SGID) on murine RAW264.7 macrophages.^46^ This hydrolysate elevated endogenous antioxidant glutathione (GSH) levels in *tert*-butylhydroperoxide (tBOOH)-treated cells and attenuated ROS in H_2_O_2_-challenged RAW264.7 cells. Table 2 summarizes the sources, the peptide sequences and the *in vitro*/*in situ* antioxidant activities of fish protein derived antioxidative peptides. Results from *in vitro* studies showed that peptide composition appears to influence their antioxidative properties. For example, peptides rich in hydrophobic amino acids demonstrate potent antioxidant properties by interacting with lipid molecules and scavenging lipid-derived radicals. GSTVPERTHPACPDFN from Hoki frame (*Johnius belengerii*), having a molecular mass of 1801 Da, exhibited high efficacy in inhibiting lipid peroxidation and in *in vitro* scavenging various free radicals such as DPPH, hydroxyl, peroxyl and superoxide radicals.^47^ The antioxidant activity was attributed to its high content of hydrophobic amino acids such as Trp and Tyr, which have the ability to stabilize free radicals and interrupt the peroxidation process. Similarly, peptides derived from blue-spotted stingray (e.g., WAFAPA and MYPGLA)^48^ and hairtail (including QNDER, KS, KA, and AKG)^49^ also show significant *in vitro* antioxidant activities due to the presence of hydrophobic residues such as Leu, Ile, Ala, Val and Met. The presence of these hydrophobic residues enhances the peptides’ ability to interact with lipid molecules, facilitating the scavenging of lipid-derived radicals and inhibiting lipid peroxidation.^50−52^

**Table 2:** Fish Protein Derived Peptide Sequences with *in Vitro* and *in Situ* Antioxidant Activities^a^

fish source	peptide sequence	antioxidant activity	ref
Hoki—frame (*Johnius belengerii*)	GSTVPERTHPACPDFN	DPPH (EC_50_: 41.37 μM), OH-radical scavenging activity (EC_50_: 17.77 μM)	(47)
blue-spotted stingray muscle (*Dasyatis kuhlii*)	WAFAPA, MYPGLA	ABTS radical scavenging activity (EC_50_:12.6 and 19.8 μM)	(48)
hairtail muscle (*Trichiurus japonicas*)	KA, AKG, IYG	DPPH (EC_50_: 0.626–0.902 mg/mL), ABTS (EC_50_: 0.586–1.652 mg/mL), OH- (EC_50_: 1.740–2.498 mg/mL), O_2_- radical scavenging activity (EC_50_: 1.355–2.538 mg/mL)	(49)
Spanish mackerel skin (*Scomberomorous niphonius*)	PFGPD, PYGAKG, YGPM	DPPH (EC_50_: 0.72–3.02 mg/mL), ABTS (EC_50_: 0.82–1.07 mg/mL), OH- (EC_50_: 0.66–0.88 mg/mL), O_2_- radical scavenging activity (EC_50_: 0.73–0.91 mg/mL)	(169)
skipjack tuna bones (*Katsuwonus pelamis*)	GADIVA, GAEGFIF	DPPH (EC_50_: 0.52 and 0.30 mg/mL), ABTS (EC_50_: 0.44 and 0.21 mg/mL), OH- (EC_50_: 0.25 and 0.32 mg/mL), O_2_- radical scavenging activity (EC_50_: 0.52 and 0.48 mg/mL)	(61)
round scad muscle (*Decapterus maruadsi*)	KGFR	DPPH radical scavenging activity (EC_50_: 0.13 mg/mL)	(60)
black shark skin	ATVY	DPPH (62.25% inhibition), ABTS radical scavenging activity (81.05% inhibition)	(53)
Atlantic sea cucumber	TEFHLL	myeloperoxidase inhibition	(170)
sturgeon (*Acipenser schrencki*) skin	GDRGESGPA	DPPH radical scavenging activity (EC_50_, 1.93 mM)	(56)
horse mackerel skin (*Magalaspis cordyla*)	NHRYDR	DPPH and •OH radical scavenging activity	(171)
croaker skin (*Otolithes ruber*)	GNRGFACRHA	DPPH and •OH radical scavenging activity	(171)
tuna dark muscle (*Thunnus tonggol*)	LPTSEAAKY and PMDYMVT	DPPH (79.6 and 85.2% inhibition) radical scavenging activity and ferric thiocyanate method	(172)
cutlassfish muscle (*Trichiurus lepturus*)	FSGE	DPPH (EC_50_: 0.03 mg/mL), eroxyl radical scavenging activity (EC_50_: 0.02 mg/mL)	(173)
stone fish flesh (*Actinopygalecanora*)	GVSGLHID	DPPH (EC_50_: 4.14 mg/mL) and ABTS radical scavenging activity (EC_50_: 3.28 mg/mL)	(174)
sea squirt (*Halocynthiaroretzi*) protein	LEW, MTTL, YYPYQL	DPPH (EC_50_:1.29–10 mM), ABTS, ORAC, Fe^2^ + chelating activity (9.20–12.5%)	(54)
tuna roe	ICRD, LCGEC	DPPH radical scavenging activity HaCaT cells: SOD, Mn-SOD, Cu-SOD, GSH-Px, MDA	(62)
tuna trimmings	ACGSDGK, KFCSGHA	myeloperoxidase inhibition, ORAC (0.82 and 0.96 μM trolox/mg peptide)	(57)
squid head	REGYFK	DPPH and ABTS scavenging activity	(175)
spotless smooth hound cartilage (*Mustelus griseus*)	GAERP, GEREANVM, AEVG	DPPH (EC_50_: 0.87–3.73 mg/mL), ABTS (EC_50_: 0.05–1.0 mg/mL), OH- (EC_50_: 0.06–0.34 mg/mL), O_2_- radical scavenging activity (EC_50_: 0.09–0.33 mg/mL) HepG2 cells: lipid peroxidation inhibition	(63)
redlip croaker—scales (*Pseudosciaena polyactis*)	GPEGPMGLE, EGPFGPEG, GFIGPTE	DPPH (EC_50_: 0.37–0.59 mg/mL), OH^–^ (EC_50:_ 0.32–0.45 mg/mL), O_2_^–^ radical scavenging activity (EC_50_: 0.47- 0.74 mg/mL). HepG2 cells: ROS level, lipid peroxidation inhibition	(64)
Hoki—skin (*Johnius belengerii*)	HGPLGPL	DPPH (EC_50_: 156.8 μM), O_2_^–^ radical scavenging activity (EC_50_: 28.8 μM). Hep3B cells: SOD (92.8% increase), GPx (60.78% increase), CAT (35% increase)	(65)
monkfish muscle (*Lophius litulon*)	EDIVCW, MGPVW, YWDAY	DPPH (EC_50_: 0.39–0.62 mg/mL), OH- (EC_50_:0.32–0.61 mg/mL), O_2_^–^ radical scavenging activity (EC_50_: 0.48–0.94 mg/mL). HepG2 cells: ROS level, lipid peroxidation inhibition	(66)
Pacific herring muscle (*Clupea pallasii*)	LHDELT, KEEKFE	DPPH (EC_50_: 5.14 and 4.37 mg/mL), OH^–^ radical scavenging activity (EC_50_: 4.57 and 3.78 mg/mL)	(67)
		HepG2 cells: cellular antioxidant activity (EC_50_: 1.19 and 1.04 mg/mL)	
Pacific cod skin gelatin (*Gadus macrocephalus*)	TCSP, TGGGNV	RAW 264.7 cells: intracellular free radical scavenging activity	(176)

a DPPH, 2,2-diphenyl-1-picrylhydrazyl; ABTS, 2′-azino-bis (3-ethylbenzothiazoline-6-sulfonic acid); ORAC, oxygen radical absorbance capacity; SOD, superoxide dismutase; GSH-Px, glutathione peroxidase; CAT, catalase; ROS, reactive oxygen species; MDA, ,malondialdehyde; H_2_O_2_, hydrogen peroxide; O_2_^–^, superoxide anion; OH, hydroxyl. One letter amino acid code used for peptide sequences.

Aromatic amino acids further contribute to antioxidant activity by stabilizing scavenged radicals. Peptides containing aromatic residues, such as Phe, Trp, and Tyr, exhibit enhanced *in vitro* radical scavenging capabilities.^52^ For instance, ATVY from black shark skin showed strong ABTS radical and DPPH radical scavenging ability due to its aromatic amino acid content.^53^ Similarly, sea squirt-derived peptides, including LEW, YYPYQL, and MTTL, displayed high antioxidant activities largely attributable to their aromatic residues.^54^ The presence of Trp, Tyr and Pro in these peptides plays a crucial role in their high antioxidant potential by donating protons to electron-deficient radicals.^55^ The aromatic amino acid residues in peptides from sturgeon skin collagen (e.g., GDRGESGPA)^56^ and tuna (including ACGSDGK and KFCSGHA)^57^ aid in stabilizing radicals and in enhancing their antioxidant properties. Moreover, the presence of Phe, an aromatic amino acid, in FSGE peptides from cutlassfish (*Trichiurus lepturus*) muscle may have contributed to the antioxidant activity.^54^

Polar amino acids are essential for hydroxyl radical scavenging and metal ion chelation.^58,59^ Peptides such as KGFR from round scad^60^ and GDRGESGPA from sturgeon skin collagen^56^ are notable for the presence of polar residues, including Lys and Arg, which contribute to their strong antioxidant and metal chelation activities. Similarly, polar AAs, including Lys, Glu, Asp and Gly, in peptides from hairtail,^61^ demonstrate the importance of polar residues in enhancing antioxidant efficacy.

Several *in situ* cell studies (Table 2) have demonstrated the ability of fish derived peptides to enhance endogenous antioxidant enzyme systems, to scavenge ROS and to protect cells from oxidative damage.^62−67^ It is important to note that although *in vitro* assays provide a controlled experimental environment, cellular bioassays involve maintaining cells outside the living organism, necessitating careful interpretation of results due to the inherent complexity of organ systems *in vivo*.

The antioxidant activity of two peptides, ICRD and LCGEC, derived from tuna roe has been evaluated in HaCaT cells from human adult skin keratinocytes.^62^ Both ICRD and LCGEC improved antioxidant activity by increasing the level of SOD and GSH-Px, by decreasing the level of MDA and by regulated the Keap1/Nrf2-ARE pathway in the cells.^62^ The presence of Cys in the peptide sequence and the low molecular mass of peptides (<3 kDa) could also be related to their antioxidant activity.^68^

Peptides derived from a tryptic hydrolysate of hoki (*Johnius belengerii*) skin gelatin were examined in cultured human hepatoma cells (Hep3B).^65^ The antioxidative enzyme levels in cells were increased in the presence of this peptide and it was presumed to be the peptide mainly involved in maintaining the redox balance in the cell environment. The N-terminally located His residue of the peptide was expected to act as a strong proton donating residue in the sequence. Furthermore, this peptide is rich in Leu, Gly, and Pro and repeats in the sequence.^65^ Similarly, three peptides (GPEGPMGLE, EGPFGPEG, and GFIGPTE) from redlip croaker (*Pseudosciaena polyactis*) scales, including Gly and Pro in their sequences, showed significant protective effects in HepG2 cells from H_2_O_2_-induced oxidative damage. They were able to decrease the levels of ROS and MDA while increasing intracellular antioxidant enzymes, including SOD, CAT and GSH-Px.^64^ Furthermore, peptides from Pacific cod (*Gadus macrocephalus*) skin gelatin, TCSP and TGGGNV, exhibited potent *in situ* antioxidant activity. The presence of hydrophobic amino acids in these peptides (Gly and Pro) and Cys could be related to their antioxidant ability.^67^

The antioxidant activities of three peptides (EDIVCW, MGPVW, YWDAY) derived from a monkfish (*Lophius litulon*) muscle protein hydrolysate was assessed in H_2_O_2_-treated HepG2 cells.^66^ These three peptides were capable of concentration-dependently protecting HepG2 cells from oxidative damage induced by H_2_O_2_. The hydrophobic/aromatic AA residues in the sequences of EDIVCW, MEPVW, and YWDAW appeared to play a crucial role in their antioxidant activity. Moreover, three peptides (GAERP, GEREANVM, AEVG) isolated from spotless smooth hound (*Mustelus griseus*) cartilage enhanced the endogenous antioxidant defense systems in HepG2 cells, including the antioxidant enzyme defense and the GSH systems.^63^ The results suggested that the presence of acidic and basic AAs, and hydrophobic AAs such as Pro and Met contributed to their antioxidant activities.

Generally, the precise mechanism responsible for the antioxidant activity of bioactive peptides has not been fully elucidated. The AA residues associated with antioxidant activity can be categorized into three groups: hydrophobic, aromatic and charged AAs. Hydrophobic and aromatic AAs are believed to function as hydrogen donors, transferring electrons to scavenge free radicals. Basic and acidic AA residues with metal chelating properties can inhibit oxidation by scavenging ferrous ions and by disrupting the oxidation chain.^69,70^ Moreover, the specificity of the hydrolytic protease and the extent of protein hydrolysis, which directly impacts the molecular mass of the hydrolysate/peptide, the AA sequence, hydrophobicity and the charge of the resulting peptides also affects the overall antioxidant activity. Peptides with molecular masses ranging from 500 to 1500 Da have generally been associated with high levels of antioxidant activity.^71,72^

The antioxidant effects of bioactive peptides derived from fish sources have been investigated in some animal studies offering a direct approach for investigating the biological mechanisms underlying the antioxidant activity induced by bioactive peptides. The peptide KTFCGRH from croaker (*Otolithes ruber*)^73^ exhibited antioxidant properties in Wistar rat resulting in increased levels of CAT, SOD and glutathione-*S*-transferase (GST) activity.

Another peptide, WHKNCF RCAKCGKSL (WL15) from snakehead fish (*Channa striatus*, *C. striatus*), demonstrated strong free radical scavenging activity in DPPH, ABTS, superoxide anion radical and hydrogen peroxide scavenging assays at 50 μM compared with the standard antioxidants Trolox and ascorbic acid. For the *in vivo* assessment, zebrafish embryos were treated with the WL15 peptide (50 μM) which attenuated the expression of activated caspase 3 expression, reduced the malondialdehyde (MDA) level and enhanced antioxidant enzyme activity, specifically that of SOD and CAT. Furthermore, the gene expression of antioxidant enzymes such as GST, GPx and γ-glutamyl cysteine synthetase (GCS) was also found to be upregulated.^70^

Moreover, collagen oligopeptides purified from tilapia scales (1000 mg/kg BW) significantly increased SOD, CAT and GSH-Px activities along with reducing the MDA content compared to the negative control group in ethanol-induced gastroduodenal injury in Wistar rats after 30 days.^74^

Through these *in vivo* trials, it was observed that studies on bioactive peptides derived from FPHs can provide valuable insights into their potential as natural antioxidants through enhancing endogenous antioxidant defense by combating oxidative stress-related damage in living organisms. However, further investigation using *in vivo* studies, particularly involving human subjects, is warranted to confirm the antioxidant effects of FPHs. To the best of our knowledge, studies focusing on the antioxidant activity of FPH-derived peptides in humans are currently lacking. This gap highlights the need for such research to provide a more comprehensive understanding of the potential antioxidant benefits of FPHs within the complex physiological processes occurring *in vivo*.

### 3.2 Glycaemic Management Effects

Type 2 diabetes mellitus (T2DM), which accounts for approximately 90% of all cases of diabetes, is currently one of the fastest growing health problems worldwide.^75^ Dietary protein, protein hydrolysates, peptides and AAs have been shown to improve glycaemic control, with their impact varying depending on the primary sequence of the peptides and AAs produced during digestion.^76−78^ Their antidiabetic activity can be via regulating blood glucose by inhibiting enzymes such as α-amylase, α-glucosidase and dipeptidyl peptidase-IV (DPP-IV), by promoting insulin signaling and via the AMP-activated protein kinase (AMPK) signaling pathway.^79^ The AMPK signaling pathway plays a multifaceted role in diabetes. It promotes glucose control by enhancing glucose uptake and utilization, improves insulin sensitivity, and protects β cells from stress and cell death.^80^ These effects can occur in various tissues, including muscle, adipose tissue and liver, each playing a distinct role in glucose metabolism and insulin sensitivity. These effects collectively contribute to the management and prevention of diabetes, providing potential therapeutic targets for the management/treatment of this metabolic disorder.

FPHs have shown the ability to enhance glucose uptake *in vivo* thereby presenting their potential for the management of hyperglycaemia alongside conventional drug therapy.^81^ These hydrolysates may ameliorate glucose tolerance either by promoting glucose uptake through a mechanism distinct from insulin or by enhancing insulin responsiveness in target cells.^81^ Collagen, for example, is an abundant protein that can be extracted from common fish coproducts such as skin, scales and bones.^82^ Pro–Hyp–Gly is the most frequently occurring tripeptide (10.5%) in collagen.^83^ Collagen and its hydrolysates/peptides has found extensive application for diabetes management.^84^

Enhancements in glycaemic management parameters due to FPHs are reported to occur via hormonal mechanisms (including the stimulation of insulin secretion), via the inhibition of DPP-IV activity to extend the duration of the endogenous incretin hormone effects and via promotion of the release of incretin hormones such as glucagon-like peptide-1 (GLP-1) and glucose-dependent insulinotropic polypeptide (GIP).^85^ Harnedy et al.^86^ and Parthsarathy et al.^87^ reported that FPHs can induce an increase in insulin secretion in BRIN-BD11 cells. Furthermore, both studies observed a significant increase in plasma insulin level in healthy mice.^86,87^ A similar increase in plasma insulin in mice was also observed by Iba et al.^88^ The plasma levels of insulin and active GLP-1 were assessed 15 min following glucose administration to male C57BL/6J mice (8 weeks old). Notably, the preadministration of fish (*Oreochromis sp.*) collagen hydrolysates 45 min prior to a glucose challenge led to an enhancement in glucose-triggered insulin secretion.^88^ Various studies have investigated GLP-1 secretion, both *in vitro* and *in vivo* following treatment with FPHs and their associated peptides.^89−91^ The chronic administration (30 days) of a tilapia skin gelatin hydrolysate resulted in a substantial increase in active plasma GLP-1 in streptozotocin-induced diabetic rats.^91^ The specific mechanism by which fish derived proteins and protein hydrolysates or peptides exert antidiabetic effects remains unclear. It remains to be investigated if the heightened level of hormone release induced by FPH arises from further peptide breakdown into free amino acids or if the peptides *per se* in the hydrolysates retain their bioactivity upon passage through the gastrointestinal tract.

While numerous investigations have explored the *in vitro* effectiveness of FPHs as inhibitors of DPP-IV,^92−97^ there exists a limited number of *in vivo* studies dedicated to assessing plasma DPP-IV activity.^98,99^ Furthermore, while many studies have demonstrated a decrease in blood glucose levels, direct measurement of plasma DPP-IV activity has often not been carried out. Some of the observed effects might indeed be attributed to reduced DPP-IV activity, however, this potential mechanism does not appear to have been directly measured during *in vivo* FPH studies, warranting further investigation.

A promising body of research indicates the potential of FPHs to enhance glucose uptake and diminish lipid accumulation *in vitro*.^86^ Amplified glucose uptake holds significance in managing blood glucose levels in T2DM, given the heightened hepatic glucose production and subsequent elevated blood glucose, which can lead to glucotoxicity and eventual α-cell dysfunction.^100^ However, a detailed understanding of the mechanisms governing glucose uptake as mediated by FPHs remains limited. Key regulators of glycemic control, including insulin receptor (IR), insulin receptor substrate-1/2 (IRS-1/2), phosphoinositide-3-kinase (PI3K) and protein kinase B (Akt) all contribute to increasing insulin sensitivity. However, no studies with FPHs appear to have been conducted thus far to investigate the upregulation of these pathways, nor have changes in insulin sensitivity been reported in any *in vivo* studies.

While a considerable amount of research has focused on the glycaemic management potential of unfractionated FPHs, there remains a relatively limited focus on the individual peptides that constitute these complex mixtures. Unearthing and characterizing the bioactive peptides present within these hydrolysates holds substantial promise for their potential ability in disease prevention and management because they may exhibit heightened bioactivity compared to the more complex hydrolysate. Studies reporting on the potential glycaemic management ability of fish derived peptides are summarized in Table 3.

**Table 3:** Fish Protein Derived Peptide Sequences with Impacts on *in Vitro* and *in Vivo* Markers of Glycaemic Management^a^

		activity		
fish source	peptide sequence	*in vitro* DPP-IV inhibitory activity (IC_50_ μM)	*in vivo*	ref
salmon (*Salmo salar*) skin	TKLPAVF, YLNF	242 and 147	Caco-2 cell monolayer membrane	(101)
salmon milt (*Oncorhynchus keta*)	LP, IP, FP	2.7	postprandial hypoglycemic effects (oral starch tolerance) in Sprague–Dawley rats.	(104)
salmon frame	IPVE, IVDI, VAPEEHPTL, IEGTL		increased glucose uptake (17%) in L6 myocytes, decreased hepatic glucose production in FaO rat hepatocytes (20%) and decreased inflammation	(108)
Atlantic salmon (*Salmo salar*)	GPAE, GPGA	49.6 and 41.9		(105)
halibut (*Hippoglossus stenolepis*) and tilapia (*Oreochromis niloticus*)	SPGSSGPQGFTG, GPVGPAGNPGANGLN, PPGPTGPRGQPGNIGF, IPGDPGPPGPPGP, LPGERGRPGAPGP, GPKGDRGLPGPPGRDGM	65.4 to 146.7		(91)
boarfish muscle (*Capros aper*)	IPVDM	21.72	Caco-2 cell: DPP-IV inhibitory activity (44.6 μM), BRIN-BD11 cells: stimulation of the insulin secretion	(107)
Atlantic salmon skin (*Salmo salar*)	LDKVER	128.71		(103)
sturgeon (*Acipenser schrencki*) skin	GPAGERGEGGPR, SPGPDGKTGPR	2140 and 2610		(56)
tilapia (*Oreochromis niloticus*) byproducts	DLVDK, PSLVH, LKPT, VAPEEHPT, DLDL, MDLP, VADTMEVV, DPLV, FAMD, CSSGGY, GPFPLLV		Caco-2 cell, DPP-IV inhibitory activity; STC-1 cell, stimulation CCK and GLP-1 secretion, lowered the blood glucose level in mice, enhanced glucose uptake into C2C12 cells	(110)
Alaska pollack (*Theragra chalcogramma*)	QWR		lowered the blood glucose level in mice, enhanced glucose uptake into C2C12 cells	(113)

a DPPIV, dipeptidyl peptidase-IV; IC_50_, half-maximum inhibitory concentration; GLP-1, glucagon-like peptide-1; CCK, cholecystokinin. One letter amino acid code used in peptide sequences.

The DPP-IV inhibitory activity of peptides sourced from salmon skin collagen by employing a combined approach involving simulated digestion and the Caco-2 cell monolayer membrane model has been investigated.^101^ Computational analysis revealed the potential DPP-IV inhibitory nature of TKLPAVF and YLNF. Assessment of their DPP-IV inhibitory potency resulted in IC_50_ values of 242.10 ± 3.40 and 146.90 ± 4.40 μM for TKLPVAF and YLNF, respectively. Molecular docking analysis highlighted the formation of seven hydrogen bonds between YLNF and DPP-IV residues, suggesting YLNF’s prospective role as a novel DPP-IV inhibitory peptide. Remarkably, YLNF showed the ability to traverse the Caco-2 cell monolayer membrane in an intact form, with an apparent permeability coefficient of 3.54 ± 0.34 × 10^–6^ cm s^–1^ at 5 mM.^101^ The presence of Phe at the C-terminal of the peptide sequence is a characteristic often associated with DPP-IV inhibitory peptides.^102^ Additionally, the presence of Thr at the N-terminus and Leu at the second position has been recognized as contributing to the DPP-IV inhibitory properties of peptides.^101^ In another study, a peptide from an Atlantic salmon (*Salmo salar*) skin collagen hydrolysate, LDKVFR, had an *in vitro* DPP-IV IC_50_ of 128.71 μM.^103^ Through molecular docking analysis, it was determined that the inhibition of DPP-IV by LDKVFR was facilitated by six hydrogen bonds and eight hydrophobic interactions between the peptide and DPP-IV.^103^ Moreover, among the peptides derived from a sturgeon (*Acipenser schrencki*) skin protein hydrolysate, two peptides demonstrated the most potent *in vitro* DPP-IV inhibitory activity compared to others. Specifically, GPAGEGEGGPR and SPGPDGKTGPR exhibited DPP-IV IC_50_ values of 2140 and 2610 μM, respectively.^56^ Molecular docking analysis further revealed that the DPP-IV inhibitory effect of these two peptides primarily arises from the formation of hydrogen bonds and hydrophobic interactions.^56^

A common approach in these studies was the use of molecular docking analysis to reveal how peptides inhibit DPP-IV activity. In the salmon skin collagen studies, peptides demonstrated their inhibitory capability through the formation of multiple hydrogen bonds with the enzyme. Similarly, in the sturgeon-derived peptides study, the peptides appear to exhibit their potent inhibitory action through the formation of hydrogen bonds and hydrophobic interactions. These findings suggest a shared mechanism across different fish-derived peptides, where hydrogen bonding and hydrophobic interactions are crucial in achieving effective DPP-IV inhibition.

A hydrolysate derived from chum salmon (*Oncorhynchus keta*) milt exhibited potent inhibitory activity against DPP-IV.^104^ Additionally, the hypoglycaemic effects of the salmon milt peptides (SMPs) were validated through oral starch tolerance tests conducted on Sprague–Dawley rats. Specifically, rats administered with SMPs at 300 mg/kg body weight over the course of 1 week experienced a notable reduction in blood glucose level 60 min after starch intake compared to the control group. Among the identified peptides, VPI and IPI showed the strongest DPP-IV inhibitory activities, with IC_50_ values of 2.7 μM.^104^ Another investigation reported that peptides derived from Atlantic salmon skin gelatin, i.e., GPAE and GPGA, showed *in vitro* dose-dependent inhibition effects on DPP-IV with IC_50_ values of 49.6 and 41.9 μM, respectively.^105^ Both peptides contain Pro as the second residue from the N-terminus, with Ala and Gly positioned adjacent to the Pro residue. Furthermore, these peptide sequences predominantly consisted of hydrophobic AAs, i.e., Ala, Gly and Pro, except for one peptide where the C-terminal residue contained the charged AA, Glu. This arrangement seems to be associated with their DPP-IV inhibitory activity.^106^

Moreover, research into collagen peptides from halibut and tilapia revealed a range of DPP-IV inhibitory activities, with IC_50_ values indicating moderate to strong inhibition. Six peptides SPGSSGPQGFTG, GPVGPAGNPGANGLN, PPGPTGPRGQPGNIGF, IPGDPGPPGPPGP, LPGERGRPGAPGP, and GPKGDR LPGPPGRDGM were identified from collagen type I α-2 and α-3 from halibut (*Hippoglossus olivaceus*) and collagen type I α-1 and α-3 from tilapia (*Oreochromis niloticus*).^91^ The *in vitro* DPP-IV IC_50_ values of these peptides ranged from 65.4 to 146.7 μM. The presence of Pro as the second residue from the N-terminus in peptide sequences may be associated with their activity, although this observation warrants further investigation. IPVDM derived from a boarfish (*Capros aper*) protein hydrolysate showed potent inhibitory activity against DPP-IV with an IC_50_ of 21.72 ± 1.08 μM in an *in vitro* assay and 44.26 ± 0.65 μM in a cell-based (Caco-2) DPP-IV inhibition assay. Additionally, this peptide significantly stimulated insulin secretion (1.6-fold increase compared to control) in cultured pancreatic BRIN-BD11 cells.^107^ Among the food protein-derived peptides, this particular peptide ranks as the third most powerful DPP-IV inhibitor (IC_50_ of 21.72 ± 1.08 μM in an *in vitro* assay) discovered, following IPI and VPL with IC_50_ values of 3.2 and 15.8 μM, respectively.^102^ The presence of Ile and Pro at positions 1 and 2, along with Val (which shares a similar structure to Ile) at position 3, was associated with the robust DPP-IV inhibitory activity.^107^ A common structural feature observed in these peptides is the presence of Pro at or near the N-terminus. This characteristic seems to play a crucial role in their ability to inhibit DPP-IV, as evidenced by the peptides described to date from chum salmon, Atlantic salmon, halibut, tilapia and boarfish.

Thirteen peptides from salmon co-products (frames) were identified, chemically synthesized, and tested for their antidiabetic bioactivities. IPVE increased glucose uptake by muscle cells (L6 myocytes), IVDI and IEGTL decreased hepatic glucose production (HGP) of insulin, whereas VAPEEHPTL decreased HGP under both basal conditions and in the presence of insulin in FaO cells from rat hepatoma.^108^ The peptides identified in this investigation primarily consist of hydrophobic AAs such as Ala, Gly, Ile, Leu, Pro, and Val. Previous research has highlighted the tendency for the AA residues in antidiabetic peptides to be predominantly hydrophobic.^109^ In fact, a blend of hydrophobic AAs, notably including Ile (2 mM) and supplemented by Cys, Met, Val, and Leu, exhibited the ability to enhance glucose uptake under both basal and insulin-stimulated conditions in isolated rat epitrochlearis muscle.^109^ Furthermore, Leu exhibited its impact on glucose metabolism through its ability to promote glycogen synthesis by deactivating glycogen synthase kinase-3 in L6 cells.^109^

Furthermore, 11 novel biopeptides were isolated from a tilapia coproduct protein hydrolysate.^110^ Among these peptides, three (DLVDK, PSLVH, LKPT) exhibited the ability to stimulate hormonal regulation of CCK and GLP-1 in Caco-2 cell. Moreover, eight peptides (VAPEEHPT, DLDL, MDLP, VADTMEVV, DPLV, FAMD, CSSGGY, GPFPLLV) demonstrated DPP-IV inhibitory activity after successfully passing through the intestinal barrier of a Caco-2 cell monolayer.^110^ This study also showed the significance of the intestinal barrier’s role in biostability, highlighting its impact on the bioactivity and peptide uptake. DLVDK and PSLVH consist of five AA residues, with some of them incorporating an aliphatic side chain. These results supported the role that the presence of an aliphatic side chain may play a significant role in stimulating CCK seretion in STC-1 cells.^111,112^

It was observed that a trypsin-digested Alaska pollack protein displayed a reduction in blood glucose levels in KK-Ay mice, a model of type II diabetes.^113^ ANGEVAQWR from a specific HPLC fraction chosen based on its glucose-lowering activity in an insulin resistance test using ddY mice was isolated. Intraperitoneal administration of ANGEVAQWR (3 mg/kg) led to a decrease in blood glucose level. Among its constituent peptides, the C-terminal tripeptide (QWR, 1 mg/kg), exhibited blood glucose-lowering effects, highlighting the importance of the C-terminal segment for this activity. Furthermore, QWR enhanced glucose uptake into C2C12 cells, a mouse skeletal muscle cell line via an insulin-independent mechanism, and decreased blood glucose levels in diabetic mice.^113^

Besides *in vitro* and *in situ* studies, human intervention studies have shown the positive impacts of bioactive proteins derived from fish in the management of T2DM through reductions in fasting blood glucose, hemoglobin A1c (HbA1c), as well as an increase in the Homeostatic Model Assessment of Insulin Resistance (HOMA-IR).^114,115^

The impact of varying doses of a cod protein hydrolysate (CPH) on postprandial glucose metabolism in older human adults has been explored as impaired glucose regulation affects a significant portion of this population.^114^ A double-blind crossover trial was conducted, where participants were administered daily doses of CPH at 10, 20, 30, or 40 mg/kg body weight for 1 week with one-week washout periods in between. The results did not demonstrate significant differences in the estimated maximum values of glucose, insulin or GLP-1 between the lowest dose (10 mg/kg BW) and higher doses (20, 30, or 40 mg/kg BW) of CPH. However, a trend was observed where higher doses of CPH seemed to lead to lower serum glucose and insulin levels.^114^ Furthermore, the effects of fish protein supplementation on glucose and lipid metabolism in thirty-four overweight adults was investigated in an 8-week randomized trial.^115^ The fish protein intake began at 3 g/d for the initial 4 weeks and increased to 6 g/d for the subsequent 4 weeks. Notably, 8 weeks of fish protein supplementation led to improved fasting glucose level, reduced 2-h postprandial glucose level and decreased glucose-area under the curve (AUC) value in comparison to the control group. Additionally, the glucose-AUC showed a significant decrease after 8 weeks with fish protein supplementation compared to baseline. Noteworthy benefits also included a reduction in LDL-cholesterol (*P* < 0.05) and favorable changes in body composition, with increased muscle percentage and reduced body fat percentage (*P* < 0.05) after 4 weeks of supplementation.^115^ The potential long-term implications of incorporating fish protein into diets warrant further exploration, especially for individuals with low glucose tolerance. Moreover, a double-blind crossover trial involving middle-aged to elderly healthy participants was conducted to examine the impact of CPH ingestion on postprandial glucose metabolism.^116^ Two separate studies were conducted with a 4- to 7-day interval between them. The participants ingested test samples containing CPH and casein as a control (20 mg/kg body weight) prior to a breakfast meal. The results indicated no discernible distinction in glucose concentration or GLP-1 concentration among the treated groups. However, the postprandial insulin concentration notably decreased in the CPH group when compared to the control group.^116^ The impact of FPH derived from blue whiting muscle when administered at doses of 1.4 and 2.8 g on various aspects including body weight, body composition and the stimulation of CCK and GLP-1 secretion has been assessed.^117^ The study involved 120 slightly overweight participants, consisting of 25% males and 75% females. Interestingly, FPH intake correlated with increased levels of CCK and GLP-1 in the blood over 90 days. However, there was no significant difference observed in these parameters between the two different doses of FPH.^117^

While significant progress has been made through *in vitro* and animal studies, the number of human studies reported to date remains limited, particularly studying the potential effects of fish protein derived peptides. Further research is needed to fully understand the benefits of these peptides for effective disease management. Gaining a deeper understanding of the bioaccessibility and bioavailability of these peptides to develop effective hypoglycaemic compounds/functional foods and evaluating the *in vivo* activity and stability of these peptides are key areas for future exploration.

### 3.3 Muscle Health

Muscle protein is in a constant state of turnover, meaning that protein synthesis is occurring continuously to replace protein lost because of protein breakdown. An abundant availability of all EAAs is a requisite for a significant stimulation of muscle protein synthesis (MPS).^118^ The BCAAs, Leu, Ile, and Val represent three of the nine EAAs. Leucine is not only a precursor for MPS, but may also play a role as a regulator of intracellular signaling pathways that are involved in the process of protein synthesis.^119^ MPS will be limited by the lack of availability of any of the EAAs, whereas a shortage of NEAAs can be compensated for by increased *de novo* production of the deficient NEAAs.^118^

Skeletal muscle mass is an important body tissue contributing to strength, performance (in sports and daily activities) and metabolic regulation. Several factors, such as calorie deficiency, resistance exercise and aging may affect the MPS to muscle protein breakdown (MPB) ratio. MPS is regulated by the mammalian target of rapamycin (mTOR) pathway.^120^

Resistance exercise increases both MPS and MPB, and when a protein-rich meal is given shortly after an exercise session, a net positive protein balance occurs^121,122^ resulting in muscle growth over time.^123−125^ MPS rates, on the other hand, are reduced and a net negative protein balance occurs during times of energy deficiency^126,127^ and as people get older^128−130^ resulting in muscle mass loss. However, it has recently been demonstrated that eating a high-protein diet can help to prevent muscle loss during periods of energy deficit and during aging^131−134^ thereby preventing/slowing down the development of sarcopenia in the elderly. Muscle plays an important role in locomotion, force production, glucose disposal^135^ and metabolic regulation.^136^ Muscle loss or low muscle level increases the risk of chronic diseases such as metabolic syndrome, type II diabetes and cardiovascular disease.^137,138^ This is also associated with increased risk of falls^139,140^ and the inability to perform daily activities,^141,142^ all of which may lead to a lower quality of life. As a result, maintaining muscle mass throughout one’s life is critical for optimum performance and general health.

According to epidemiological research, the elderly are at a higher risk of not consuming enough high quality protein. Over the age of 50, 32–41% of women and 22–38% of men consume less protein than recommended (RDA for proteins 0.8 g kg^1^ per day), and nearly no older adult consumes the highest Acceptable Macronutrient Distribution Range (AMDR) for protein (35% of total energy intake).^143^ The decreased ability of skeletal muscle in older adults to respond to anabolic stimuli could be caused by several factors. These may include a reduction in physical activity levels,^144^ increased retention of AAs in the splanchnic area (particularly L), resulting in diminished levels of AAs in the bloodstream,^145^ a state of chronic low-level inflammation,^146,147^ decreased expression of AA transporters in skeletal muscle^148,149^ and weakened anabolic signaling pathways.^150,151^

Seafood is gaining popularity among consumers and proponents of healthy living because of its high quality protein, polyunsaturated fatty acids, trace mineral and vitamin content.^152^ Seafood’s highly digestible and high-quality proteins offer the majority of the body’s required and EAAs for muscle health^153^ as well as energy.^154^ Furthermore, the existence of bioactive peptides, which may be released during gastrointestinal digestion, is thought to be primarily responsible for the bioactive or biofunctional actions of proteins.^14^ Bioactive peptides contain sequences of AAs with functional groups that contribute to their biological activity, e.g., having hormone like activity or the ability to modulate the activity of key metabolic pathway enzymes.^155^

The presence of high-quality protein and a diverse AA profile in blue whiting (*Micromesistius poutassou*) prompted an investigation into the potential impacts of three distinct blue whiting protein hydrolysates (BWPHs) on the growth, proliferation and MPS of skeletal muscle myotubes (C2C12).^156^ The production of the BWPHs involved varying enzymatic and heat exposures, followed by simulated gastrointestinal digestion (SGID), resulting in different degree of hydrolysis (DHs) (ranging from 33.4 to 37.3%) and substantial proportions of low molecular mass peptides. Muscle growth and myotube thickness were evaluated employing an xCelligence platform, revealing that the BWPHs significantly promoted both parameters compared to the negative control (amino acid and serum-free media) (*p* < 0.01 for muscle growth and *p* < 0.0001 for myotube thickness). Furthermore, MPS, as measured by puromycin incorporation, was significantly higher after incubation with one of the BWPHs compared with the negative control.^156^ Moreover, the impact of an SGID-treated sprat (*Sprattus sprattus*) protein hydrolysate (SPH) on the growth, proliferation and MPS in skeletal muscle (C2C12) myotubes was examined. The SGID-SPH significantly increased myotube growth and thickness compared to the negative control (cells grown in AA and serum-free medium). Puromycin incorporation was also significantly higher after incubation with SPH-SGID compared with the negative control (*p* < 0.05) indicating the potential ability of SPH to stimulate MPS.^157^ These *in situ* studies provide evidence regarding the beneficial impact of fish protein on skeletal muscle hypertrophy and its underlying mechanisms.

Furthermore, several human studies assessed the effects of consuming fish protein hydrolysates on physical performance and muscle mass.^158−163^Table 4 summarized the outcomes of these studies. The role of protein supplementation in conjunction with exercise is addressed by a comprehensive study conducted by Vegge et al., who investigated the potential ergogenic effects of unprocessed whey protein and a hydrolyzed marine protein supplement known as NutriPeptin (Np), in combination with carbohydrate supplementation.^158^ The study involved trained male cyclists and employed a double-blinded crossover design. Results showed that ingestion of unprocessed whey protein (15.3 g·h^–1^) along with carbohydrate (60 g·h^–1^) did not exhibit any significant impact on 5 min mean-power performance after a 120 min cycling session at 50% of maximal aerobic power. In contrast, the hydrolyzed marine protein supplement (Np) (2.7 g·h^–1^) when combined with a protein-carbohydrate beverage (PROCHO) (12.4 g·h^–1^ and 60 g·h^–1^), demonstrated an ergogenic effect on mean-power performance.^158^ However, another randomized, double-blind crossover design study involved 12 healthy males was conducted in three experimental trials.^159^ These trials comprised a 90 min cycling task at 50% of predetermined maximum power output, followed by a 5 km time trial (TT). The nutritional interventions were administered at 15 min intervals during the cycling task, encompassing CHO, CHO-PRO (a combination of carbohydrate and whey protein), and CHO–PRO-PEP (incorporating hydrolyzed marine peptides along with carbohydrate and whey protein). It was reported that while the addition of hydrolyzed marine peptides did influence metabolism, as evident from metabolic responses, the ultimate endurance exercise performance, evaluated through a 5 km TT, did not exhibit significant differences across conditions. Therefore, while the presence of hydrolyzed marine peptides seemed to exert an influence on metabolic responses, it did not confer a discernible ergogenic advantage when assessed in the context of 5 km TT performance.^159^

**Table 4:** Human Intervention Trials Exploring the Effects of Fish Proteins/Hydrolysates on Skeletal Muscle Health, Function, and Exercise Performance

marine protein source	participants	research design	key findings	ref
Nutripeptin (Np; codfish-based)	well-trained male cyclizts (age 22 ± 2 years)	randomized, double-blind, crossover trial	5 min mean-power did not differ between groups; blood urea nitrogen significantly increased in treatment group	(158)
salmon	healthy aerobically trained males, age 23 years	randomized, double-blind, crossover design	mean 5 km TT time to completion and power output did not differ between trials	(159)
Nile tilapia (*Oreochromis niloticus*)	physically active subjects (6 males, 3 females; age 27 ± 2 years)	randomized, double-blind, placebo-controlled, crossover trial	rapid and pronounced amino acidemia was observed	(160)
Atlantic cod fillet (*Gadus morhua*)	86 participants, 57 women and 29 men. 65 years old and older	randomized, double-blind, control trial	no difference was found between the intervention and control groups in grip strength and gait speed	(161)
fresh frozen meat from Atlantic cod	14 healthy male volunteers	a double-blinded crossover	there were no significant differences between the two nutrition supplementations measured by time to exhaustion at the cycling sessions	(162)
blue whiting (*Micromesistius poutassaou*)	7 healthy older adults (two males, five females; age: 72 ± 5	randomized, counterbalanced, double-blind design	blue whiting protein hydrolysate (BWPH) induced postprandial essential amino acidemia in older adults above the control; insulin was elevated above baseline in BWPH; myotube hypertrophy was greater in BWPH compared with control	(163)

Another study aimed to unravel the distinctive postexercise amino acidemia patterns following the consumption of a whey protein hydrolysate (WPH) (0.25 g/kg) and an FPH (0.25 g/kg) in a double-blind, randomized crossover design cohort of nine physically active individuals (six males and three females).^160^ The results revealed significant elevations in plasma concentrations of TAA, EAA, BCAA, and L at 30 to 60 min following FPH supplementation, as well as at 30 to 120 min following WPH intake compared to the control. No significant differences emerged in plasma TAA, EAA, BCAA, and L concentrations between FPH and WPH at any time point.^160^ Collectively, these findings highlight the parallel and rapid postexercise amino acidemia triggered by both FPH and WPH, positioning FPH as a promising alternative source of rapidly digested proteins for postresistance exercise utilization. A double-blinded, randomized, controlled trial was conducted to assess the potential benefits of a marine protein hydrolysate (MPH) supplementation (hydrolysate of fresh or fresh-frozen Atlantic cod (*Gadus morhua*) fillet) (3 g of protein per day) on physical function and strength among 86 elderly individuals during 12 months.^161^ No significant differences were found between the intervention and the control groups in terms of the mean change in short physical performance battery (SPPB) or the temporal trend in SPPB, grip strength or gait speed. It was concluded that the participants, notably characterized by their relatively high functional status, possibly encountered a ceiling effect in the SPPB measurement. Additionally, their adequate protein intake and engagement in physical activity might have influenced the outcomes.^161^ Similarly, another randomized controlled study with cross over design investigated the potential benefits of incorporating FPH in a supplement alongside whey protein (WP) and carbohydrate (CHO) on short-term recovery following high-intensity performance.^162^ Fourteen healthy male cyclists were engaged in a double-blinded crossover design study consuming nutrition supplementation either containing MPH (hydrolysate of fresh or fresh-frozen Atlantic cod (*Gadus morhua*) fillet) or excluding MPH (CHO-WP-MPH or CHO-WP), followed by a 4-h recovery period.^162^ There were no significant distinctions observed between the two nutrition supplementations in terms of time to exhaustion, heart rate, respiratory exchange ratio, blood lactate concentration and glucose levels during the high-intensity performance cycling sessions. This suggests that the incorporation of MPH in the protein supplement failed to exert discernible effects on short-term recovery, when compared to supplementation without MPH.^162^ While the study’s relatively brief recovery period could be considered a limitation, it may also present a potential advantage for MPH. Protein supplements, including MPH, might offer enhanced benefits in terms of protein synthesis and glycogen replenishment, particularly when recovery time is limited.^162^

Furthermore, the effects of a protein hydrolysate sourced BWPH on AA levels in the body and muscle growth in C2C12 mouse muscle cells, comparing it with whey protein isolate (WPI) and a control containing nonessential amino acids (NEAA) were assessed.^163^ The study design involved both *ex vivo* and *in vitro* approaches, utilizing blood samples from older adults and treating muscle cells with serum-conditioned media. While both BWPH and WPI triggered essential AA increase in the serum of older adults, this increase was more pronounced with WPI. Insulin levels showed a greater increase with WPI and BWPH compared to the NEAA control. Muscle protein synthesis was enhanced in serum from WPI-fed participants, and it was significantly enhanced on ingestion of WPI and BWPH when compared with ingestion of NEAA. The muscle cells also exhibited greater hypertrophy with WPI and BWPH than with the NEAA.^163^

These findings underline the potential of FPHs to enhance muscle mass and function, particularly among older adults. While fish-derived proteins hold promise in promoting muscle adaptation, their effects can be influenced by factors such as recovery time and individual functional status. These insights collectively emphasize the significance of fish protein supplementation and dietary interventions in tackling age-related muscle loss. However, the diverse outcomes across studies also underscores the need for further research, considering factors such as protein source, dosage, individual variability and specific exercise protocols. Moving forward, the existing data supports a role for fish proteins/hydrolysates in the improvement of muscle health and offers valuable insights into designing effective nutritional strategies to address muscle health and performance in aging populations.

Certain peptides derived from food proteins may promote the accumulation of skeletal muscle protein, indicating their potential as beneficial agents for muscle health enhancement. Protein hydrolysates, which contain di- and tripeptides, have been shown to alleviate sarcopenia.^164^ Compared to free AA and intact protein, protein hydrolysates are more efficiently absorbed, with bioactive peptides exhibiting high specificity, efficacy, selectivity, biocompatibility and low immunogenicity.^163,165,166^ Investigating the effect of fish-derived peptides on muscle health is of significant importance for several reasons. First, fish is known for its high-quality protein content, including bioactive peptides that can support muscle health by providing EAAs necessary for muscle growth, maintenance and repair. These peptides can contribute to muscle recovery and repair by potentially possessing anti-inflammatory properties, aiding in tissue repair and reducing inflammation. Furthermore, fish-derived peptides may stimulate muscle protein synthesis through specific amino acid sequences, activating signaling pathways involved in muscle growth and promoting the production of new muscle proteins. Additionally, these peptides may exhibit antioxidant and anti-inflammatory activities, safeguarding muscle tissue from the damage caused by oxidative stress and chronic inflammation. Considering muscle wasting conditions, such as cancer, diabetes, and aging-related disorders, fish-derived peptides with potential anabolic or anticatabolic effects could have therapeutic applications in preserving muscle mass and in mitigating muscle loss.

This review examined the multifaceted roles of FPHs and peptides focusing on their antioxidant properties, glycaemic management capabilities and muscle health benefits. Analysis of the various *in vitro*, *in situ*, and *in vivo* studies published to date indicates the potential applications of these bioactive compounds. It is worth mentioning that while *in vitro* and small animal *in vivo* studies have provided valuable information, translating these findings to human health requires comprehensive human intervention trials. In addition, there is a need for further well-designed and controlled human studies involving larger and more diverse populations. This review shows that the mechanisms through which FPHs and specific peptide sequences exert their effects on various physiological processes may be complex and multifaceted, necessitating a more in-depth exploration to establish causality and to elucidate the underlying pathways. Therefore, in order to validate and support the outcomes observed in the current studies, a robust body of evidence from rigorously conducted human intervention trials is essential. Such studies would provide a clearer understanding of the potential benefits, appropriate dosages and the safety profiles of FPHs/peptides in human nutrition and in health promotion.

## Abbreviations

AA: amino acid
TAA: total amino acid
EAA: essential amino acid
NEAA: nonessential amino acid
BCAA: branch chain amino acid
FPH: fish protein hydrolysate
CPH: cod protein hydrolysate
WPH: whey protein hydrolysate
MPH: marine protein hydrolysate
WPI: whey protein isolate
BWSPH: blue whiting soluble protein hydrolysate
APP: Alaska pollock protein
APF: Alaska pollock frame
SGID: simulated gastrointestinal digestion
ROS: reactive oxygen species
BHA: butylated hydroxyl anisole
DPPH: 2,2-diphenyl-1-picrylhydrazyl
ABTS: 2,2′-azino-bis(3-ethylbenzothiazoline-6-sulfonic acid)
ORAC: oxygen radical absorbance capacity
SOD: superoxide dismutase
CAT: catalase
GSH-PX: glutathione peroxidase
GST: glutathione *S*-transferase
tBOOH: *tert*-butyl hydroperoxide
TBARS: thiobarbituric acid reactive substances
PUFA: polyunsaturated fatty acid
MPO: myeloperoxidase
MDA: malondialdehyde
EC_50_: half-maximal effective concentration
IC_50_: half-maximal inhibitory concentration
T2DM: type 2 diabetes mellitus
DPP-IV: dipeptidyl peptidase-4
GLP-1: glucagon-like peptide-1
GIP: gastric inhibitory polypeptide
CCK: cholecystokinin
HB: hemoglobin
MPS: muscle protein synthesis
MPB: muscle protein breakdown
MyHC: myosin heavy chain
SPPB: short physical performance battery
